# Why invest in Research & Development for sorghum and millets? The business case for East and Southern Africa

**DOI:** 10.1016/j.gfs.2020.100458

**Published:** 2020-09

**Authors:** A. Orr, C. Schipmann-Schwarze, A. Gierend, S. Nedumaran, C. Mwema, E. Muange, E. Manyasa, H. Ojulong

**Affiliations:** a ICRISAT, Nairobi, Kenya; b ICRISAT, Hyderabad, India; c Machakos University, Kenya; d WorldFish, Zambia; e University of Kassel, Germany

**Keywords:** Sorghum, Millets, Africa, Commercialization

## Abstract

This article synthesizes recent research by ICRISAT and its partners to analyse the business case for sorghum and millets in ESA and the wider strategy of commercialization on which this is based. The business case is stronger for sorghum because of its greater impact on poverty and food security, but millets are better suited to a strategy of commercialization. Commercial demand for millets is primarily driven by specialty markets for flour while that for sorghum is limited to beer. Demand for improved varieties is driven primarily by the need for early – maturity that shortens the hungry period. Future growth in production depends on increased opportunities for inter-regional trade.

## Highlights

• Synthesis of recent unpublished research by ICRISAT.
• Production rising after long decline.
• Commercial demand also growing but still limited.
• Adoption of improved varieties not driven solely by commercialization.
• R & D strategy should emphasise benefits to household food security over cash income.

## 1 Introduction

At first sight the business case seems strong. Sorghum (*Sorghum bicolor*) and millets (*Pennissetum glaucum, P. typhoides, P. tyhpideum, P. americanum* and *Eleusine coracana*) are grown by tens of millions of smallholder farmers in South Asia and Sub-Saharan Africa (SSA) (ICRISAT & Partners, 2017). 1 They are grown primarily in the semi-arid and sub-humid agro – ecologies (Gumma et al., 2017) where their adaptability to high temperatures and drought (Hyman et al., 2016) makes them resilient to climate change (Adhikari, 2015). One-third of the population in these areas live below the international poverty lines of $1.25 per day (Gumma et al., 2017). For many poor households, therefore, sorghum and millets are a vital source of food security, particularly in drought years when other cereal crops may fail.

Yet public investment in research and development (R & D) has become increasingly hard to justify. Global production of sorghum has fallen from 66 million (m) t in the period 1980–82 to 60 m t in 2016–18. In the same period, the production of millets has flatlined, from 26 m t to 29 m t (FAOSTAT, 2020). India, once the world's biggest producer of sorghum, has seen output slump by almost one-third (Bhagavatula et al., 2013). By contrast, in the same period global production of maize rose by from 431 m t to 1146 m t (FAOSTAT, 2020). The perception that sorghum and millets cannot compete with other cereal crops weakens the business case for R & D. If production of sorghum and millets is declining worldwide, why invest scarce research resources?

Africa is different, however. In the years 1980–82 and 2016–18, the production of sorghum in SSA doubled from 13 to 29 m t, while the production of millets increased from 8 to 14 m t (FAOSTAT, 2020). This reflects differences in the demand drivers for these crops. In India, rising income, changing consumer preferences, and subsidized prices for rice and wheat have resulted in declining demand for sorghum and millets as staple food crops, with half of production now going to alternative uses such as poultry feed and raw material for the alcohol and food processing industries (Bhagavatula et al., 2013).In SSA, by contrast, sorghum and millets are still primarily staple food crops (Orr et al., 2017).

These contrasting trends highlight the need for a regional approach to investment in R & D. This article focuses on East and Southern Africa (ESA), where the production of sorghum has doubled between 1980–82 and 2016–18 to reach 8 m t, while the production of millets has risen from 1.3 to 1,8 million t. East Africa's 16 countries 2 are geographically diverse, ranging from the highlands of Ethiopia to the Rift Valley region in Kenya and the large uncultivated regions of Tanzania. This complex topography has produced a semi-arid/arid climate with a bimodal pattern of rainfall. Southern Africa comprises five countries. East Africa has six times as many poor people – 75 m living below the international poverty line of $1.25 per day – as Southern Africa, which has only 12 m (Gumma et al., 2017).

R & D for sorghum and millets in Africa is led by the International Crops Research Institute for the Semi-Arid Tropics (ICRISAT), one of the 15 International Agricultural Research Centers (IARCs) that make up the Consultative Group for International Agricultural Research (CGIAR). ‘ICRISAT’ here means ‘ICRISAT and partners’, including National Agricultural Research Systems (NARS), Non-Government Organisations (NGOs) and private firms. ICRISAT's business case for sorghum and millets is embedded in a wider R & D strategy that centers on commercialization:

*“[S]orghum and pearl millet producers are caught in a subsistence production trap. The lack of a commercial market for these crops encourages farmers to maintain a subsistence level of technology and production. Yet the development of a commercial market is discouraged by the lack of a consistent marketable surplus. (*Rohrbach and Kiriwaggulu, 2007*: 5).*

To break out of this trap, ICRISAT's R & D strategy focuses on raising productivity, arguing that only higher yields can achieve the increase in supply that is needed to make these crops more competitive and stimulate commercial demand. Simultaneously, raising productivity depends on linking farmers with markets, because only markets can give farmers the incentive to adopt improved varieties and invest in improved crop management.

The general objective of this article **i**s to analyse the business case for R & D for sorghum and millets. Specifically, we analyse six supporting arguments:

1. Demand for sorghum and millets is growing;
2. Demand is being driven by commercialization;
3. Commercialization is also driving adoption of improved varieties;
4. Adoption of improved varieties will reduce poverty and hunger;
5. Sorghum and millets will increase resilience to climate change; and
6. The return on investment in R & D is high.

This article is based on a synthesis of unpublished research by ICRISAT carried out between 2011 and 2017 by Phase I of the Harnessing Opportunities for Productivity Enhancement (HOPE) project. However, while these studies provide a wealth of information on individual topics, the ‘big picture’ is missing. In this article, we try to fill this knowledge gap by synthesising the results from 20 of these studies, structured around the evidence for the business case for R & D. Although this article is not based on a comprehensive literature review, selective use has been made of other studies where these provide additional relevant information.

The article is organised as follows. The next section describes the data and methods. Section 3 presents the evidence for the six arguments supporting the business case. Section 4 discusses these results and their implications for ICRISAT's R & D strategy. The final section concludes.

## 2 Data and methods

A ‘business case’ may be defined as a set of reasoned arguments, backed by quantitative evidence, to justify a particular investment. Ideally, a business case includes (1) the strategic justification for the investment (2) the appraisal of different options (3) the expected benefits (4) the commercial costs and benefits (5) the potential risks involved and (6) the time required for the delivery of these benefits. This article addresses numbers (1), (3) and (6). Unlike a commercial seed company where the expected benefits are dictated by profitability (4), for ICRISAT the business case is founded primarily on the contribution that R & D can make to wider development goals (3) of reduced rural poverty, improved food security, improved nutrition and health (ICRISAT and partners, 2017).

### 2.1 Data

Table 1 shows the sources of data used in this study. Collectively, the evidence covers the entire spectrum from adoption and crop production to processing, value chain development and impacts. Of these 20 reports, 14 are based on primary data, including household surveys (Nos. 7, 8, 9, 10, 11, 18, 19, 20), national household expenditure surveys (No. 4), interviews with consumers (No. 5) and processing companies (No. 3), and on-station trials (No. 12) and crop modeling (Nos. 13, 14). Six reports are based largely on secondary data, including value chain development (Nos. 2, 6), poverty impacts of crop improvement (Nos. 15, 16, 17) and foresight analysis (No. 1). All but two of these reports were prepared by ICRISAT and 16 are available online as SocioEconomics Discussion Papers (ICRISAT, 2018).

**Table 1:** Reports used in this synthesis.

No.	Subject	Crop	Topics	Countries	Reference Period/Year	Data and methods	Sample size	Reference
1	Foresight analysis	Sorghum and millets	Demand, climate change, commercialization	ESA	2015–2050	FAO statistics, IMPACT model		Orr et al. (2016).
2	Demand	Sorghum, millets	Flour, clear beer, animal feed	Kenya, Ethiopia, Tanzania, Uganda	2015–2025	Secondary data		Orr et al. (2017)
3	Demand	Sorghum, millets	Flour processing industry	Kenya, Tanzania, Uganda	2013	Company interviews	25 companies (Tanzania), 15 companies (Uganda), 13 companies (Kenya)	Schipmann-Schwarze et al. (2015).
4	Demand	Sorghum, millets	Consumer demand for grain and flour	Kenya, Tanzania, Uganda, Ethiopia	2005–2012	National household expenditure surveys	13,430 (Kenya), 10,463 (Tanzania), 6775 (Uganda), 21,595 (Ethiopia),	Gierend and Orr (2015).
5	Demand	Sorghum, millets	Consumer demand and preferences for processed products	Kenya	2012	Consumer interviews	454 consumers (Kenya), 439 consumers (Tanzania)	Schipmann et al. (2012).
6	Value chain development	Sorghum, millets	Warehouse receipt system, contract growing	Kenya, Uganda	2013	Secondary data		Orr and Mwema (2013).
7	Value chain development	Sorghum	Clear beer, social inclusion	Kenya	2012–13	Household survey	300 households	Orr and Mwema (2013).
8	Production and utilization	Sorghum, millets	Adoption, production, utilization	Tanzania	2010	Household survey	360 households	Schipmann et al. (2014).
9	Production and utilization	Finger millet	Adoption, production, utilization	Uganda	2015	Household survey	190 households	Mwema et al. (2017)
10	Production and utilization	Finger millet	Adoption, profitability, marketing, social inclusion	Kenya	2012	Household survey	270 households	Handschuch (2014)
11	Production and utilization	Sorghum, pearl millet	Adoption, profitability, utilization	Mozambique	2014	Household survey	142 households	Tsusaka et al. (2015).
12	Improved crop management	Sorghum, millets	Fertilizer, weeding, tied ridging	Kenya, Tanzania	2010–11	On-station trials		Mwema and Orr (2014).
13	Improved crop management	Sorghum	Plant population, fertilizer, tied ridging, mulching, intercropping	Tanzania		Crop modeling		Dixit (2012).
14	Improved crop management	Sorghum	Plant population, fertilizer, tied ridging, mulching, intercropping	Ethiopia		Crop modeling		Dixit (2013)
15	Crop improvement	Sorghum	Return on investment, poverty impact, poverty mapping	Tanzania	1980–2030	Secondary statistics, DREAM model		Gierend et al. (2014a).
16	Crop improvement	Sorghum, millets	Return on investment, poverty impact, poverty mapping	Uganda	1965–2030	Secondary statistics, DREAM model		Gierend et al. (2014b).
17	Crop improvement	Sorghum	Return on investment, poverty impact, poverty mapping	Ethiopia	1971–2040	Secondary statistics, DREAM model		Gierend et al. (2014c).
18	Adoption	Sorghum	Improved seed	Tanzania	2010	Household survey	360 households	Schipmann-Schwarze et al. (2014)
19	Adoption	Sorghum	Improved seed, social networks	Tanzania	2012	Household survey	345 households	Muange and Schwarze (2014)
20	Impact	Sorghum	Adoption, profitability, welfare	Tanzania	2012–13	Household survey	822 households	Kaliba (2014).

### 2.2 Methods

Space does not allow a full description of the methods used to collect and analyse this data. Full details are given in the individual reports themselves. The salient features are summarized below:

#### 2.2.1 Foresight analysis

Information on projected trends to 2050 was obtained from the International Model for Policy Analysis of Agricultural Commodities and Trade (IMPACT) (Orr et al., 2016). In this model, supply is determined by crop area, prices and the rate of productivity growth. These crop- and country-specific growth rates summarize the improvements that can be achieved in agricultural productivity from advances in management practices, crop improvement and agricultural extension. Supply, demand, and price relationships for each commodity are based on elasticities derived from country-level studies. The model simulates the operation of national and international markets, solving for production, demand, and prices that equate supply and demand across the globe. The effect of trade policies has also been included in the model to reflect the price differential between country and world prices. When supply exceeds demand in a country or region, the difference goes into a box called ‘net trade’, with countries and regions being either net importers or exporters.

Four scenarios were modelled:

1. A ‘baseline’ scenario where income and population growth were set to medium level, with no climate change;
2. An ‘optimistic’ scenario where population growth was set to a low level and income growth to a high level, with no climate change;
3. An ‘increased yield’ scenario where productivity growth rates of sorghum, millets and maize were increased by 25% above the baseline level; and
4. A ‘climate change’ scenario where the impact of two climate models, the Geophysical Fluid Dynamics Laboratory – Earth System Model 2 (GFDL-ESM2M) and the Model for Interdisciplinary Research on Climate – Earth System Model – Chemistry (MIROC-ESM-CHEM) – representing the driest and wettest models – on the production of sorghum and millets were compared to the baseline scenario, without climate change. Each crop is 'grown” first with 2000 climate and then with 2050 climate, with identical location-specific inputs. Irrigated crops are assumed to receive as much water as needed so irrigated crop yield effects are driven by temperature only. Yield effects for rainfed crops combine both temperature and precipitation effects.

#### 2.2.2 Commercialization

Information on demand for six value chains derives from secondary statistics at the national level for Ethiopia, Tanzania, Uganda, and Kenya (Orr et al., 2017). Information on flour processing was obtained from interviews with 53 milling companies in Kenya, Tanzania, and Uganda in 2012 (Schipmann-Schwarze et al., 2015). Information on consumer demand derived from nationally – representative household expenditure surveys conducted in Ethiopia, Tanzania, Uganda, and Kenya between 2005 and 2012 (Gierend and Orr, 2015). Information on consumer preferences was obtained by a sample survey of consumers in rural and urban areas of Kenya and Tanzania in 2012 (Schipmann-Schwarze et al., 2012).

#### 2.2.3 Commercialization and adoption

Information on comparative rates of commercialization and adoption was obtained from household surveys in central Tanzania (Schipmann et al., 2013), eastern and western Kenya Kenya (Orr et al., 2013; Handschuch, 2014), northern Uganda (Mwema et al., 2017) and central Mozambique (Tsusaka et al., 2015). These rates are not nationally – representative but based on small samples in selected areas. Commercialization was measured as the share of total harvest sold. Since information on the share of crop area planted to improved varieties was not available from all surveys, adoption was measured as the share of farmers adopting improved varieties.

#### 2.2.4 Improved crop management

Information on the expected benefits from improved varieties and crop management practices for sorghum was obtained from crop models using the Agricultural Production Systems sIMulator (APSIM) crop model (Dixit, 2012, 2013). The models were calibrated based on rainfall and soils data for Dodoma (central Tanzania) and Melkassa (central Ethiopia). Results from research trials for sorghum in Kenya and Tanzania and for finger millet in Kenya provided further information on profitability (Mwema and Orr, 2014).

#### 2.2.5 Return on investment

The rate of return on investment for R & D was measured using the Dynamic Research EvaluAtion for Management (DREAM) model (Gierend et al., 2014 a,b,c), based on workshops with plant breeders in Tanzania, Uganda and Ethiopia. Breeders estimates were modified using sensitivity analysis. The results for Tanzania are based on a 20% increase in yield from improved varieties (Gierend, *pers. comm*.). Those for Ethiopia are based on breeders' yield estimates, reduced by 50% (Gierend et al., 2014c). For Uganda, sensitivity analysis showed little difference when based on a 20% increase in yields or on breeders' yield estimates. These yield increases are based on the increase expected on small, well-managed fields and may be higher than the yield which an ‘average’ farmer would obtain if they adopted improved varieties.

#### 2.2.6 Impact on poverty

The impact of R & D on poverty was measured comparing the share of households below the national poverty line based on ‘basic needs’ with the share of benefits from R & D captured by ‘poor’ farmers (Gierend et al., 2014 a,b,c). Research gains were based on the original estimates made by breeders rather than on the revised estimates we used to measure returns to investment (see 2.2.5). However, this did not significantly affect the relative share of gains between the ‘poor’ and ‘non-poor’.

#### 2.2.7 Impact on household food security

Information on days to maturity for improved varieties is available for the region (Mgonja et al. (2005), Monyo et al. (2002), and from the national registers of improved crop varieties for Kenya (KEPHIS, 2018), Tanzania (Kanyeka et al., 2007), Uganda (2015). and Ethiopia (EIAR, 2004; MOANR, 2016). Missing or new information was supplied by ICRISAT plant breeders.

## 3 Results

Because the available data does not cover all the countries in ESA, only the results for the demand and climate change model projections (Table 2 and Fig. 2) cover the region as a whole. Other results – on consumption, utilization, commercialization, poverty, food security, and returns to investment – refer only to the specific countries named in the relevant Table or Figure.

**Table 2:** Production, demand and trade projections for sorghum and millets, Eastern and Southern Africa, 2015–2050 (000 t).

Variable	Sorghum			Millets		
	Scenario: “Baseline”					
,	2015	2025	2050	2015	2025	2050
Production	11,782	16,004	29,899	3177	4517	8961
Demand	12,008	15,634	26,307	3431	4445	7280
Net Trade	−1328	−731	2490	−254	72	1681

	*Scenario: “Optimistic”*					
Production	11,405	15,101	27,036	3133	4383	8549
Demand	10,308	12,923	21,278	3500	4420	7186
Net Trade	−4	1077	4657	100	539	2236

	*Scenario: “25% increase in yield growth rate of sorghum and millets”*					

Production	12,166	17,072	34,917	3312	4976	11,312
Demand	12,026	15,691	26,605	3435	4458	7347
Net Trade	−952	280	7211	−123	513	3965

	*Scenario: “25% increase in yield growth rate of maize”*					

Production	11,896	16,124	29,775	3170	4497	8858
Demand	11,768	15,347	25,857	3431	4444	7274
Net Trade	−1379	−823	2086	−261	53	1584

Source: IMPACT Model. For individual countries see Orr et al. (2016): Table 6.1–6.7.

Note: ESA is defined following the FAOSTAT classification. Eastern Africa: Burundi, Djibouti, Eritrea, Ethiopia, Kenya, Madagascar, Malawi, Mauritius, Mozambique, Rwanda, Somalia, Sudan, Uganda, Tanzania, Zambia, and Zimbabwe, while Southern Africa is defined as Botswana, Lesotho, Namibia, South Africa, and Swaziland. This differs from the definition for ESA used by the IMPACT model (Orr et al., 2016, Appendix 1).

### 3.1 Demand projections

Foresight analysis showed that demand for sorghum and millets in ESA is growing. Assuming ‘business as usual’ with current rates of income and population growth, demand for sorghum in ESA is projected to reach 29.9 m t by 2050, up from 11.8 m in 2015 (an increase of 153%) (Table 2). Demand for millets is also projected to grow by 178%, but from a lower base, from 3.2 m t in 2015 to 8.9 m t in 2050. Although slower population growth and faster growth in income (the ‘optimistic scenario’) will reduce demand, nevertheless demand will still increase, reaching 27 m t for sorghum and 8.5 m t for millets by 2050.

For ICRISAT's commercialization strategy, the most relevant scenario is that of ‘25% increase in yield growth of sorghum and millets’. Faster growth in yields is expected to increase supply and reduce prices, making sorghum and millets more affordable and boosting demand. However, the results from this scenario are disappointing. Demand for sorghum in ESA is projected to reach 21.3 m t 2050, while demand for millets will reach 7.2 m t, which are below the levels of demand in the ‘baseline’ or ‘business as usual’ scenario’. Since demand within the region is limited, faster yield growth increases ‘net trade’. In the ‘baseline scenario’ the volume of sorghum and millets that goes into net trade by 2050 is projected to reach 2.5 m t for sorghum and 1.7 m t for millets. Under the ‘scenario of ‘25% increase in yield growth rate for sorghum and millets', by 2050 7.2 m t of sorghum and 3.9 m t of millets would have to be exported outside the region, which is equivalent to 21% and 35% of the region's production of sorghum and millets, respectively. The scenario for a ‘25% increase in the yield growth rate of maize’ results in virtually no change over the ‘baseline scenario’, either in the production of sorghum and millets or in demand and net trade.

### 3.2 Commercialization: urbanization and ‘new uses’

Table 3 presents the evidence on consumption of sorghum and millets for four countries in ESA. Urbanization has reduced the consumption of sorghum per head, which fell from 30 kg/capita/year in rural areas to just 7 kg. Rising income had the same effect, though not as sharply. In both urban and rural areas, sorghum consumption fell with income. The exception was rural Ethiopia, reflecting geographical concentration in the ‘sorghum belt’ where it is preferred to other cereals. Consumption of millets was much lower than for sorghum, averaging just 6 kg/capita/year. Urbanization reduced average consumption of millets by just 3 kg/capita/year. In Tanzania and Uganda, urban consumption was slightly higher than in rural areas, whereas in Kenya consumption was the same. Similarly, in urban Tanzania and Uganda, consumption was greatest among high-income consumers just as in rural areas. Unlike sorghum, consumer demand for millets rose with income.

**Table 3:** Consumption of sorghum, millets, and maize in Ethiopia, Tanzania, Uganda and Kenya (kg/capita/year).

Country	Sorghum			Millets			Maize		
	By location								
	National	Urban	Rural	National	Urban	Rural	National	Urban	Rural
Ethiopia	50	14	57	9	3	11	44	11	51
Tanzania	16	8	18	6	5	7	70	46	79
Uganda	7	9	4	5	4	6	53	39	59
Kenya	3	Na.	Na.	2	2	2	94	58	109
Weighted mean	26	7	30	6	4	7	71	38	59
	Urban, by income								
	Sorghum			Millets			Maize		
	Low	Middle	High	Low	Middle	High	Low	Middle	High
Ethiopia	14	15	12	3	4	2	17	18	11
Tanzania	10	3	7	2	4	7	39	48	58
Uganda	4	1	1	4	2	6	40	37	26
Kenya	<1	<1	<1	3	2	2	52	75	60
	Rural, by income								
	Sorghum			Millets			Maize		
	Low	Middle	High	Low	Middle	High	Low	Middle	High
Ethiopia	39	61	68	7	11	13	57	75	93
Tanzania	23	13	12	5	8	20	67	104	142
Uganda	14	6	3	5	6	7	60	54	58
Kenya	<1	<1	<1	1	1	2	102	110	110

Source: Gierend and Orr (2015), p.49, Table 12; p. 54, Table 14.

Table 4 shows estimates of the utilization of sorghum and millets in six value chains in 2013 and (based on key assumptions) in 2025. At present, commercial utilization is confined to the value chains for flour (sorghum and millets) and beer (sorghum). None of the processors surveyed in Kenya used sorghum grain or bran as a source of livestock feed. Flour processing is the dominant value chain, accounting for 88% of utilization in these four countries. Sorghum can be mixed with wheat flour to produce composite wheat flour. Consumer taste preferences restrict the ratio of sorghum used to 5% (Orr et al., 2017). However, at present there is no production of composite wheat flour in ESA. The value chain for clear sorghum beer accounts for 11% of commercial utilization. Growing demand reflects the success of Senator keg, which has become Kenya's best-selling beer (Orr et al., 2013). Except in Uganda, where Nile Breweries make *chibuku*, opaque sorghum beer is brewed by the informal sector.

**Table 4:** Utilization of sorghum and millets in six value chains for Ethiopia, Tanzania, Kenya, and Uganda, 2013–2025 (000 t).

Value chain	Sorghum		Millets	
	Current utilization	Potential utilization	Current utilization	Potential utilization
	(2013)	(2025)	(2013)	(2025)
1. Sorghum and millet flour	315	347 ^1^	153	169 ^1^
	(88)	(42)	(100)	(100)
2. Composite wheat flour	0	148 ^2^	0	0
	(0)	(18)	(0)	(0)
3. Maize meal	0	94 ^3^	0	0
	(0)	(11)	(0)	(0)
4. Livestock feed	0	64 ^4^	0	0
	(0)	(8)	(0)	(0)
5. Opaque beer	2	3 ^5^	0	0
	(1)	(0)	(0)	(0)
6. Clear beer	39	69 ^6^	0	0
	(11)	(8)	(0)	(0)
Total utilization	356	823	153	169
	(100)	(100)	(100)	(100)
Available supply	6578 ^7^	6578	1432	1432
	(5)	(13)	(11)	(11)

Source: Orr et al. (2017), Table 1, Table 2.

Notes.

^1^. Assuming current urban consumption plus 10%, and successful marketing of Smart Foods.

^2^. Assuming 5% substitution of maize, 15–20% price reduction in wholesale price of sorghum.

^3^. Assuming 5% substitution of wheat, 15–20% price reduction in wholesale price of sorghum.

^4^. Assuming rate of substitution of 100% between white sorghum and maize, and a target of 10% of current utilization and 20% price reduction in wholesale price of sorghum.

^5^. Assuming past growth in beer production is sustained.

^6^. Assuming past growth in beer production is sustained, plus favourable tax regime.

^7^. Total production in 2013.

Potentially, the utilization of sorghum could expand to include the value chains for animal feed, maize meal and wheat flour. However, there are pre-conditions. First, the utilization of sorghum in these value chains requires a sorghum price 15–20% lower than that of maize or wheat. Currently, sorghum in ESA sells at a 20% price premium over maize (Gierend and Orr, 2015).. Second, the utilization of sorghum for animal feed requires better information about the nutrient composition of sorghum. Feed manufacturers in Kenya were not convinced that white sorghum can replace maize for livestock feed without loss of feed quality (Orr et al., 2017). In the case of millets, the value chain with the highest potential utilization was the value chain for flour. Utilization in other value chains is limited by the price premium for millet of 20% over sorghum and 40% over maize (Gierend and Orr, 2015).

### 3.3 Commercialization and adoption of improved varieties

**Fig. 1:**
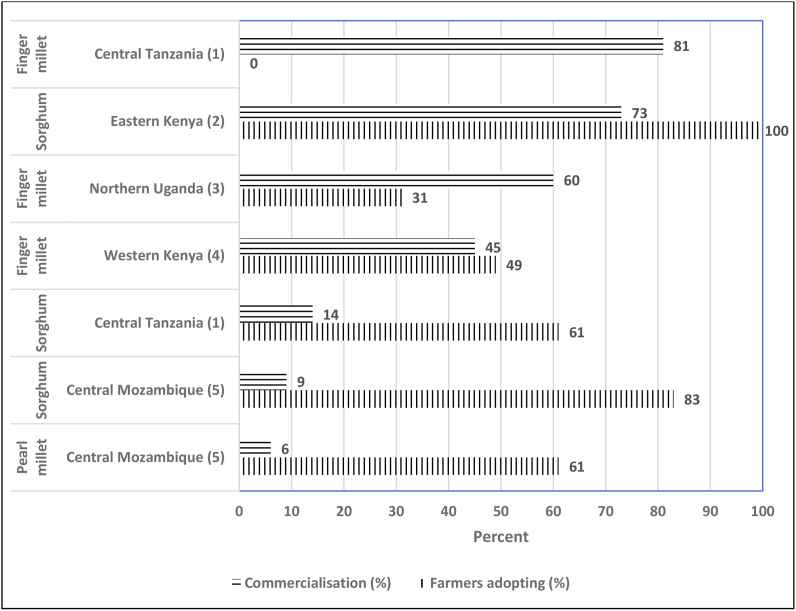
Commercialization and adoption of improved varieties (%). Sources: ^1.^Schipmann et al. (2013). ^2^Orr et al. (2013). ^3^Mwema et al. (2017). ^4^Handschuch (2014). ^5^Tsusaka et al. (2015).

Fig. 1 shows that commercialization was highest for finger millet in Tanzania (81% sold) and for sorghum in eastern Kenya (73% sold). However, while in Kenya all the sample growers had adopted an improved variety, in Tanzania the adoption rate was zero, since no improved varieties had yet been released. In contrast, the surveys in Mozambique showed low levels of commercialization for sorghum (9%) and pearl millet (6%) but relatively high levels of adoption – 83% for sorghum and 61% for pearl millet.

**Fig. 2:**
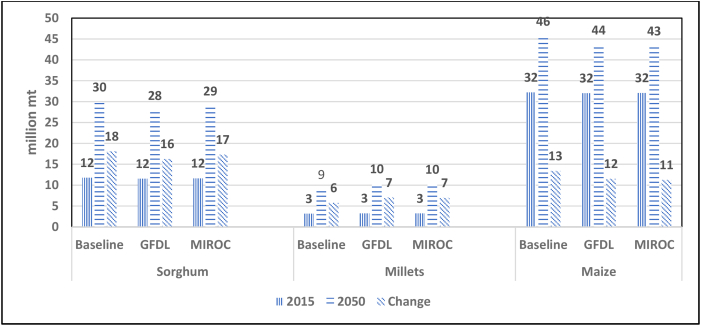
Impact of climate change on production of sorghum, millets and maize in ESA 2015–2050. Source: IMPACT model. Notes: GFDL = Geophysical Fluid Dynamics Laboratory – Earth System Model 2. MIROC = Model for Interdisciplinary Research on Climate – Earth System Model – Chemistry.

### 3.4 Climate change

Although average yields will decline, the area planted to sorghum and millets will rise as they replace less resilient crops. In the case of sorghum, both models project that production in ESA will drop slightly to 28–29 m t by 2050, compared to 30 m t in the baseline projection (Fig. 2). For millets, the models project that production will increase over the baseline projection of 9 m t to 10 m t by 2050, an increase of 11%. By contrast, the production of maize will decline by 3–7% over the baseline projection.

### 3.5 Impact on poverty

**Fig. 3:**
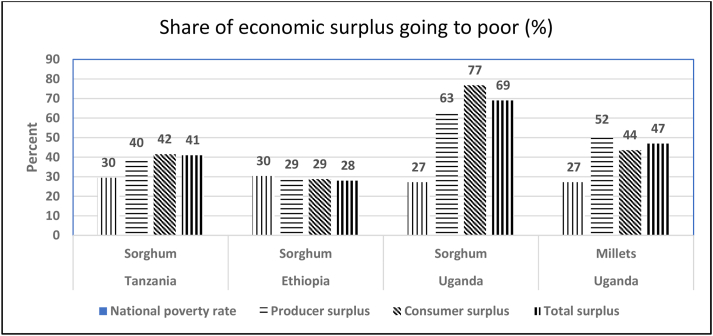
Potential impacts of R & D on poverty. Sources: Tanzania: Gierend et al. (2014b), p.64 Table 42; Ethiopia: Gierend et al. (2014c), p. 113 Table 73; Uganda: Gierend et al. (2014a), p. 72 Table 62.

Fig. 3 compares the national poverty rate for Tanzania, Ethiopia and Uganda with the share of the benefits from R & D going to ‘the poor’. For sorghum in Tanzania and Uganda and for millets in Uganda, the share of benefits to the poor exceeded the national poverty rate. The exception was Ethiopia, where the share going to the poor was close to the national poverty rate. Sorghum in Uganda was the most ‘poverty-friendly’ of the four R & D programs.

### 3.6 Impact on food security

Table 5 shows that 66% of improved sorghum varieties can be classified as ‘early’ with a minimum field duration of 90 days, or 120 days in the Ethiopian highlands, compared to 19% classed as ‘late’. The split for millets was more even – only 51% of improved varieties were ‘early’ – because of the large number of ‘late’ millet varieties released in Ethiopia.

**Table 5:** Improved varieties in Ethiopia, Tanzania, Uganda and Kenya, by category of maturity date.

Category of maturity date	Ethiopia	Tanzania	Uganda	Kenya	Total
Sorghum					
Early ^a^	8	6	6	26	46
	(35) ^g^	(86)	(100)	(76)	(66)
Medium ^b^	7	0	0	4	11
	(30)	(0)	(0)	(12)	(16)
Late ^c^	8	1	0	4	13
	(35)	(14)	(0)	(12)	(19)
All	23	7	6	34	70
	(100)	(100)	(100)	(100)	(100)
Millets					
Early ^d^	2	8	2	10	22
	(10)	(100)	(50)	(91)	(51)
Medium ^e^	2	0	0	0	2
	(10)	(0)	(0)	(0)	(5)
Late ^f^	16	0	2	1	19
	(80)	(0)	(50)	(9)	(44)
All	20	8	4	11	43
	(100)	(100)	(100)	(100)	(100)

Sources: Ethiopia: Gierend et al., 2014a, Gierend et al., 2014b, Gierend et al., 2014c, EIAR (2004), MOANR (2015); Tanzania: Kanyeka et al. (2007); Uganda: Kenya: KEPHIS (2018). General: Monyo et al. (2002).

Notes.

^a^ Sorghum: 120–130 days in Ethiopian Highlands, elsewhere 90–110 days.

^b^ Sorghum: 131–150 days in Ethiopian Highlands, elsewhere 111–125 days.

^c^ Sorghum: 150 > days in Ethiopian Highlands, elsewhere 125 > days.

^d^ Finger millet: 90–100 days; pearl millet 60–70 days.

^e^ Finger millet: 100–120 days; pearl millet: 80–90 days.

^f^ Finger millet: 120 days>; pearl millet: 90 days>.

^g^ Figures in parentheses are percentages.

### 3.7 Return on investment

Table 6 shows a return on investment (ROI) of between $ 38 and $ 59 per dollar invested. The largest total surplus was for sorghum in Ethiopia, Uganda and Tanzania, and the smallest for millets in Uganda. For sorghum in Ethiopia and Uganda, the benefits went primarily to producers (78% and 61%, respectively), while for sorghum in Tanzania they went primarily to consumers (66%). In Uganda, the benefits for millets were split evenly between producers (46%) and consumers (54%). These returns are based on the level of yields expected on farmers’ fields using recommended management practices.

**Table 6:** Return on investment in R & D for sorghum and millets in Tanzania, Ethiopia and Uganda (000 USD).

Country	Tanzania	Ethiopia	Uganda	
Crop	Sorghum	Sorghum	Sorghum	Millets
Period	1980–2030	1971–2040	1980–2030	1965–2030
Total Surplus (TS)	122,992	173,893	125,555	43,441
Producer (PS)	41,985	138,511	76,642	19,950
Consumer (CS)	81,007	35,382	48,913	23,511
Research Costs (RC)	2107	4564	2132	897
TS-RC	120,885	169,329	123,432	42,547
Return on Investment (ROI) (USD)	58	38	59	48
TS per year (USD)	2365	2415	2415	648

Source: Gierend et al. (2014a, b, c).

## 4 Discussion

### 4.1 The business case

Based on the results in the previous section, the business case for sorghum and millets can be summarized under six heads:

#### 4.1.1 Production is going up

The secular decline in the production of sorghum and millets has now been reversed. The IMPACT model predicts that between 2015 and 2050 the production of both sorghum and millets in ESA will almost treble (Table 2). True, faster growth in income and slower growth in population would slow these increases in production. of sorghum. But these effects could be cancelled out by faster growth in yields and climate change. R & D that accelerated the rate of growth in yields of sorghum and millets would result in an even greater rise in production. For both crops, faster growth in yields has a greater positive effect on future production than the effects of climate change, highlighting the importance of R & D.

One reason for this reversal is that sorghum and millets are no longer in direct competition with maize. Accelerating the growth rate of growth in the yield of maize by 25% - and holding everything else constant – has minimal impact on the production of sorghum and millets (Table 2). Sorghum and millets are now concentrated in agro-ecologies where farmers regard them as insurance crops and are unwilling to rely solely on maize for household food security. On the demand side, consumers increasingly see sorghum and millets not as substitutes for maize but as complementary. Maize is consumed as a main meal in the form of stiff porridge (*ugali*). Inside production areas, sorghum and millets may also be consumed as *ugali*, but outside these areas they are consumed as a thin porridge for breakfast (*lishe* or *uji* in Swahili) (Schipmann-Schwarze et al., 2015). Consumers prefer sorghum or finger millet flour as weaning food for children or, as with sorghum in Ethiopia, the preferred ingredient for mixing with teff (*Eragrostis tef*) to make the staple bread *injera*.

#### 4.1.2 The forces driving demand up are stronger than those driving it down

Urbanization and rising income will continue to have a negative impact on the aggregate demand for sorghum. Sorghum consumption drops from 30 kg/capita/year in the countryside to just 7 kg/capita per year in urban areas, while consumption of millets drops from 7 to 4 kg/capita/year (Table 3). Higher income consumers in both urban and rural areas also have a lower demand for sorghum. The exception is Ethiopia, where higher income rural consumers who can afford to eat *injera* reduce the cost by mixing sorghum with teff.

On the other hand, population growth and consumer preferences are driving demand up. In ESA, where population will double within 25 years, these extra mouths will increase aggregate demand for sorghum and millets, as for other cereals. Unlike sorghum, demand for millets rises with income (Table 3). Africa's growing middle-class will help drive this demand. Annual expenditure on processed food by middle-class consumers in five ESA countries is expected to top $53 billion by 2040 (Tschirley et al., 2015). Although most flour is still sold loose in open-air markets, which allows blending according to personal taste (Schipmann-Schwarze et al., 2012), among middle-class consumers there is growing demand for pre-packed flour, sold in urban supermarkets (Schipmann-Schwarze et al., 2015). Flour processors reported increasing demand for pre-packed flour and expected demand to grow, especially for finger millet flour (Schipmann-Schwarze et al., 2015). According to processors, the main constraint on this demand was the lack of consumer awareness of the nutrition and health benefits (Schipmann-Schwarze et al., 2015). One in three of consumers already buying sorghum or millets were unaware of these benefits; among non-consumers, the ratio was two in three. Consumers reported that TV and radio were the most effective channels for information on these benefits (Schipmann-Schwarze et al., 2012). Marketing sorghum and millets as ‘Smart Foods’ with health benefits for young children and for adults at risk of lifestyle diseases can accelerate the growth of demand for these flours (ICRISAT, 2019). Lastly, one should not forget policy. In ESA, reducing the excise duty on beer made from locally – sourced materials helped create demand for clear sorghum beer from lower income ‘aspirational’ consumers ‘trading-up’ from illegal brews (Orr et al., 2013), True, such policies can suddenly be reversed, with devastating results (Orr, 2018), but with rising consumer income and competition within the industry to reach new customers, the demand for clear sorghum beer seems set to grow.

#### 4.1.3 Resisting climate change

As befits drought-tolerant crops, sorghum and millets perform well against climate change. A review of different climate models concluded that, in terms of yield, sorghum is more resilient to climate change than millets and both are more resilient than maize. A drier, warmer climate in eight ESA countries may reduce the yield of sorghum by 5% and the yield of millets by 15% (Adhikari et al., 2015). However, the IMPACT simulations for ESA predict that neither the driest (GFDL-ESM2M) nor the wettest (MIROC-ESM-CHEM) climate models will have much impact on the production of sorghum and millets or maize (Fig. 2). One explanation for this result is the diversity of climatic regimes within the region, with significant variations between countries in rainfall and the length of the growing season. In some countries (eg. Ethiopia), a drier climate (the GFDL model) increases the production of sorghum while in others (eg. Sudan) production is reduced. Similarly, a wetter climate (the MIROC model) reduces the production of sorghum in Sudan but increases production in Ethiopia and Kenya. The same principle holds true for maize. Summed across the region, these changes cancel out, resulting in little change in net production at the aggregate level. Millets are clear winners against climate change, with production rising in both models. The IMPACT model assumes that farmers make no attempt to mitigate the effect of climate change. If farmers were to adopt improved management practices, such as micro-dosing with inorganic fertilizer, this would more than compensate for the loss of yield by predicted by these climate models (Cooper et al., 2009).

#### 4.1.4 Profitability through trade

A sudden jump in supply can result in a production glut and falling prices. In Uganda, for example, sorghum prices collapsed when Nile Breweries stopped buying Epuripur sorghum after just two seasons, by which time they had accumulated enough stock to last them two years (FARM-Africa, 2007). This outcome can be avoided if unwanted grain is exported. Already, Kenya's flour processors rely on imports of finger millet from Uganda (Handschuch, 2014; Schipmann-Schwarze et al., 2015). while Kenya's East African Breweries relies on imports of sorghum from Tanzania and Sudan (Orr et al., 2013). Yet Tanzania and Uganda periodically impose export bans on cereal crops. In response to high food prices, Tanzania banned the export of cereals between January 2008 and April 2010. According to Unga Mills in Nairobi, this led to a 60% rise in the purchase price of finger millet in Kenya (Orr and Mwema, 2013).

Trade is therefore essential to ensure profitability. The IMPACT model assumes that any production resulting from R & D that cannot be absorbed within the region can be exported. In the ‘baseline’ or ‘business as usual’ scenario, 8% of sorghum and 19% of millets will be traded outside the region by 2050. In a scenario where R & D accelerates the rate of growth in yields by 25%, this rises to 21% and 35%, respectively. Increasing exports of finger millet from Uganda will shift the benefits from R & D dramatically in favour of producers (Gierend, 2014a). Similarly, market integration and cross-border trade will double the benefits from R & D for sorghum in Tanzania, with gains going primarily to producers (Gierend et al., 2014b). Hitherto, sorghum and millets have been viewed exclusively in terms of food policy. These results highlight the need to view them as part of trade policy.

#### 4.1.5 Reduced poverty and hunger

In general, R & D had a favourable impact on poverty because the benefits went primarily to poorer regions. In Uganda, the impact of R & D on poverty was higher for sorghum than for millets because sorghum was concentrated in ‘poverty hotspots’ in the Northern and Eastern regions, whereas millets were concentrated in the Teso region where the poverty rate was below the national average (Gierend et al., 2014a). In Tanzania, although poverty rates in sorghum-growing regions varied, the average rate was still above the national average (Gierend et al., 2014b). By contrast, in Ethiopia there was little difference in the poverty rate at the national level and in areas where sorghum was grown. This reflected the ubiquity of sorghum as well as similar poverty rates across regions (Gierend et al., 2014c).

Commercialization can also reduce poverty if it includes poorer farmers. Collective action improves access to markets for poorer farmers. In Western Kenya, women who belonged to farmer groups were more likely to obtain higher prices for their finger millet (Handschuch, 2014). Similarly, among farmer groups in Eastern Kenya supplying the value chain for clear sorghum beer, households headed by women were twice as likely to be members as households headed by men (Orr et al., 2013). Groups give farmers information about prices and improved varieties. The lack of information on sorghum and millets from formal extension services forces farmers to rely on social networks (Handschuch, 2014; Muange and Schwarze, 2014). The bigger their network, the more likely are farmers to have adopted improved sorghum varieties (Muange and Schwarze, 2014).

R & D will also improve household food security. ICRISAT's R & D strategy assumes that the benefits to food security will come from higher yields of improved varieties (ICRISAT & Partners, 2017). But even if improved varieties did not increase yields, they would still improve household food security by accelerating the harvest. Two-thirds of improved sorghum varieties and half of improved millet varieties are classed as early-maturing (Fig. 3). In Tanzania, the most popular improved variety of sorghum, Macia, has a field duration of 110–120 days (Kaliba, 2014) and matures a full two months before Langalanga, the most popular local variety, which has a field duration of 180–210 days (Kisija, 2012). Improved varieties of sorghum have been adopted not only for their higher yields but because they mature early (Kaliba, 2014). However, early – maturing varieties of sorghum are shorter, with less straw for fodder, thatching, and fencing. This has discouraged their adoption in Ethiopia (Orr et al., 2016). In Uganda, early – maturity was second only to higher yields as the reason for adopting improved varieties of finger millet (Mwema et al., 2017).

#### 4.1.6 High returns on investment

R & D for sorghum and millets gives a high rate of return. The evidence for Tanzania, Uganda and Ethiopia shows that R & D for sorghum gave a return of between $ 38–59 for every dollar invested, while R & D for finger millet gave a return of $ 48 (Table 5). A systematic review of R & D studies confirms that R & D for sorghum and millets generates high social rates of return (Zereyesus and Dalton, 2017). On the other hand, a rate criterion must always be judged against the absolute value of the returns. On this criterion, the greatest return was for sorghum in Ethiopia ($ 174 m or $ 2415 per year). By contrast the total return for finger millet in Uganda was a mere $ 6 million or $ 648 per year. Improved varieties of finger millet that are resistant to blast disease (*Magnaporthe grisea),* which is found throughout the region, are an international public good that might justify investment by ICRISAT (Lenné et al., 2007). However, the CGIAR's centre-wide Research Program (CRP) recently discontinued funding for finger millet in ESA (*pers. comm*. Henry Ojulong).

### 4.2 Implications for ICRISAT's R & D strategy

While the results generally support the business case, they also challenge three assumptions underlying ICRISAT's strategy of commercialization.

#### 4.2.1 Commercialization will drive demand

In specific areas, commercialization drives the demand for millets. In Tanzania, Uganda and Kenya, over 45% of the crop is sold (Fig. 1). However, sorghum is primarily a subsistence crop. In Ethiopia, Eritrea, Mozambique and Tanzania less than 15% of sorghum is sold (Orr et al., 2017). The exceptions are eastern Kenya and northern Tanzania, where demand is driven by the value chain for clear sorghum beer.

Commercialization is not being driven by ‘new uses’. High prices rule out the use of millets as feed for livestock or (as in India) for poultry (Orr et al., 2017). Utilization in the value chains for clear sorghum beer, specialty flour, and livestock feed accounted for just 6% of the total current supply of sorghum and 11% of millets. The number of growers linked to new value chains was small. In 2015, the number of smallholders involved in the value chain for clear sorghum beer was estimated at 13,000, less than 1% of the total number of sorghum growers in Kenya, Kenya, Uganda, and Tanzania (Orr et al., 2017). Demand by these value chains is unlikely to increase dramatically in the foreseeable future. Potential commercial demand in 2025 was estimated at 15% of current supply for sorghum and 12% for millets (Orr et al., 2017). Sorghum's importance for food security means that households reduce sales in drought years. After poor rains in eastern Kenya, only one-third of sorghum growers contracted by Smart Logistics were willing to sell even part of their harvest (Esipisu, 2011). Processors identified inconsistent supply as a major barrier to commercialization (Schipmann-Schwarze et al., 2015).

#### 4.2.2 Commercialization will drive adoption of improved varieties

The evidence for a significant positive relationship between commercialization and adoption was mixed. In eastern Kenya, commercialization clearly drove the adoption of Gadam, an improved variety of sorghum with white grains and a low tannin content suitable for brewing clear beer (Orr et al., 2013). But the link was less clear in the value chain for flour. In northern Uganda, where 60% of finger millet was sold, only 31% of sample farmers had adopted improved varieties, while in central Tanzania, where 81% of finger millet was sold, there were no improved varieties available (Fig. 1).

One reason for this lack of relationship was that improved varieties did not necessarily give higher yields when grown in farmers’ fields. True, in Uganda (Mwema et al., 2017) and western Kenya (Handschuch, 2014) yields from improved varieties of finger millet were reported to be twice as high as from local varieties. In Tanzania, one survey reported that improved varieties of sorghum gave higher yields (Kaliba, 2014), but other surveys found no significant difference (Monyo et al., 2004; Schipmann et al., 2013). One explanation for low yields from improved varieties is that sorghum and millets are planted primarily on marginal land where they will give at least some yield in drought years but where yields in years of average rainfall are low. Higher yields may also depend on improved management practices. However, not all these practices were profitable. Crop modeling for sorghum in Tanzania and Ethiopia revealed that micro-dosing with 30 kg N ha^−1^ was profitable for all the improved varieties studied, the profitability of increasing plant populations and intercropping was mixed, but that tied ridging and mulching were unprofitable (Dixit, 2012, 2013). Chemical control was also less profitable than hand-weeding for finger millet in Kenya, suggesting the need for more effective herbicides (Mwema and Orr, 2014).

Commercialization did not always stimulate investment in cash inputs. Neither smallholders in Kenya supplying the value chain for sorghum beer nor those in Tanzania supplying the value chain for millet flour applied fertilizer (Orr et al., 2013a; Schipmann et al., 2013). Only finger millet in Western Kenya was fertilized (Handschuch, 2014). Even where markets exist, therefore, farmers may be unable to increase yields because they lack access to the credit needed to buy fertilizer. Yet even where credit is available, they may be unwilling to apply fertilizer to sorghum and millets rather than to maize, which is grown on more productive land and where fertilizer will give a higher return. As a result, improved varieties were not always more profitable. On a cash cost basis (excluding the cost of family labour), the gross margin for Gadam sorghum in eastern Kenya was just 56 USD ha ^−1^ (Orr et al., 2013). However, in Uganda and western Kenya, improved varieties of finger millet were more profitable than local varieties (Handschuch, 2014; Mwema et al., 2017).

#### 4.2.3 Commercialization requires price incentives

One option is to store grain after harvest and wait for prices to rise. However, the seasonal rise in prices was much lower for sorghum and millets than for maize, which greatly reduced the potential benefits of a Warehouse Receipt System for these crops (Orr and Mwema, 2013). A second option is to pay higher prices at harvest. Take the value chain for clear sorghum beer (Orr et al., 2013b). A private company, Smart Logistics, offered growers a price of 22 Kenyan Shillings (KES)/kg compared to 12 KES/kg paid by local buyers. This incentive price was made possible by economies of scale and by contracting directly with East African Maltings Limited which agreed to buy at a price of 30 KES/kg (Orr and Mwema, 2013). This business model was profitable because, despite a lower profit margin, the volume of sorghum sold by Smart Logistics was high (Orr et al., 2013b).

Higher harvest prices are also possible in speciality value chains that cater for a niche market. With their reputation as health foods, sorghum and millets have less need to compete on price with maize or wheat flours. Annual expenditure on processed food by middle-class consumers in five ESA countries – which includes demand for nutrient-dense sorghum and millet flour by health-conscious consumers – is expected to top $ 53 billion by 2040 (Tschirley et al., 2015). Over 70% of processors surveyed in Kenya, Tanzania and Uganda were willing to pay a price premium for clean grain, undamaged by pests (Schipmann-Schwarze et al., 2015). One-third of companies surveyed in Tanzania and Uganda, and two-thirds of processors in Kenya, had experience with contracting with smallholders. However, direct contracting was time-consuming, expensive because of the distance between farmers and processors, and unreliable because contracts could not be enforced. Consequently, processors found it easier to buy grain from private traders at the factory gate.

On the other hand, higher harvest prices are counter-productive where demand is limited and there are cheaper substitutes. In the value chains for livestock and poultry feed, the need was not for price incentives for growers but for lower wholesale prices for processors. Feed processors in Kenya reported that to increase utilization as livestock feed, sorghum would have to be at least 20% cheaper than maize (Orr et al., 2017). Similarly, to compete in the value chains for composite wheat flour and maize meal the price of sorghum would have to fall by 20% (Orr et al., 2017). However, except in Ethiopia, sorghum prices in ESA were higher than for maize and wheat (Orr et al., 2016). Increasing the demand for sorghum in this value chain would therefore require a hefty reduction in farmgate prices.

## 5 Conclusions

Our analysis reveals a paradox. Millets are well-suited to a strategy of commercialization but the business case for R & D is weaker, whereas for sorghum the business case is stronger but the crop is less well-suited to a strategy of commercialization.

The business case for R & D is persuasive. Demand for sorghum and millets is growing, driven by a high rate of population growth, which offsets the negative effects of urbanization and a fall in average consumption as incomes rise. In response, growers have increased supply, which is projected to almost treble by 2050. Higher production will reduce poverty in Tanzania and Uganda, because sorghum and millets are grown where the poverty rate is above the national average, and will be poverty-neutral in Ethiopia, where poverty is spread more evenly. Household food security will improve thanks to higher yields and a shorter hungry period. Both crops are resilient to climate change, which slightly reduces the production of sorghum but increases that for millets. Finally, the rate of return on investment from R & D is impressive. However, the *scale* of the benefits from sorghum dwarfs those from millets. In terms of ICRISAT's wider development goals, this makes the business case for sorghum stronger than for millets.

In contrast, the evidence for a strategy of commercialization is less convincing. It confirms the relevance of a ‘subsistence production trap’ for specific value chains. Sorghum's higher price limits its substitution for maize in the value chains for livestock feed, wheat flour and maize meal. Breaking out of this trap requires a supply-shift to increase yields and reduce wholesale prices. But the trap is less relevant for the value chains for specialty flours and clear sorghum beer, where commercialization is being driven by shifts in demand as the result of higher incomes and changing consumer preferences. Price incentives can kick-start commercialization in these value chains but will not benefit the value chains for maize meal, wheat flour, or livestock and poultry feed where increasing the demand for sorghum will require lower wholesale prices. Lastly, while there are examples where commercialization has driven the adoption of improved varieties, in other cases improved varieties have been adopted because they are early-maturing and shorten the hungry period. ICRISAT's R & D strategy needs not just greater realism about the prospects for commercialization but also a heavier emphasis on the benefits for household food security.

## Declaration of competing interest

The authors declare that they have no known competing financial interests or personal relationships that could have appeared to influence the work reported in this paper.

## Footnotes

1 The FAO statistics do not distinguish between different species of millet. In this article we use ‘millets’ to include both pearl millet (*Pennissetum glaucum, P. typhoides, P. tyhpideum, P. americanum*) and finger millet (*Eleusine coracana*). For their relative distribution in ESA see Orr et al. (2016).

2 FAO defines Eastern Africa as: Burundi, Djibouti, Eritrea, Ethiopia, Kenya, Madagascar, Malawi, Mauritius, Mozambique, Rwanda, Somalia, Sudan, Uganda, Tanzania, Zambia, and Zimbabwe, while Southern Africa is defined as Botswana, Lesotho, Namibia, South Africa, and Swaziland.
